# The Role of PTEN in Tumor Angiogenesis

**DOI:** 10.1155/2012/141236

**Published:** 2011-09-05

**Authors:** Stéphane Rodriguez, Uyen Huynh-Do

**Affiliations:** Department of Clinical Research and Department of Nephrology and Hypertension, Inselspital, University of Bern Medical School, 3010 Bern, Switzerland

## Abstract

During the past 20 years, the phosphatase and tensin homolog PTEN has been shown to be involved in major physiological processes, and its mutation or loss is often associated with tumor formation. In addition PTEN regulates angiogenesis not only through its antagonizing effect on the PI3 kinase pathway mainly, but also through some phosphatase-independent functions. In this paper we delineate the role of this powerful tumor suppressor in tumor angiogenesis and dissect the underlying molecular mechanisms. Furthermore, it appears that, in a number of cancers, the PTEN status determines the response to chemotherapy, highlighting the need to monitor PTEN expression and to develop PTEN-targeted therapies.

## 1. Physiological Angiogenesis

**Figure 1:**
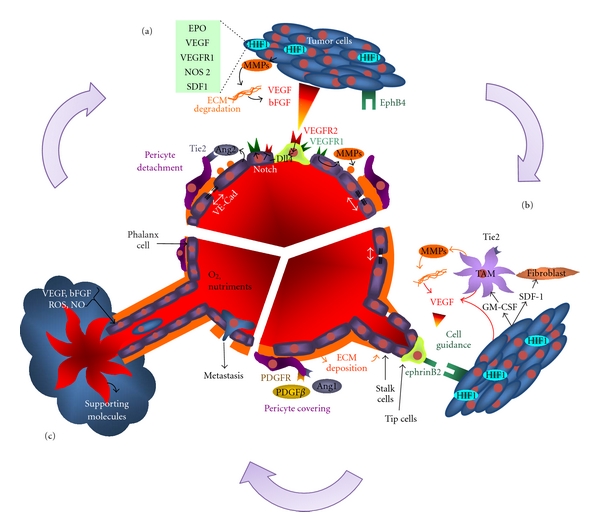
Molecular basics of tumor angiogenesis. Key players of tumor angiogenesis and their main functions are depicted here. (a) Once a tumor has reached a volume of 1-2 mm^3^, tumor cells start to be hypoxic, and HIF1*α* is stabilized. Activated HIF pathway leads to the expression of several genes (within green square) which greatly contribute to VEGF production. VEGF amplify this system through VEGF-dependent MMPs expression involved in ECM degradation and growth factors release. VEGF acts as a chemoattractant on endothelial cells from the nearest vessels and triggers vessel sprouting. Stimulated VEGFR2 leads to the expression of Dll4, a Notch ligand which inhibits the tip cell transformation through VEGFR1 upregulation. VEGFR activation mediates proteases expression, VE-Cad complex disruption leading to cell/cell and cell/matrix detachment. In parallel, Tie2 stimulation by Ang2 induces pericytes detachment. This step is required for endothelial cell migration and proliferation. (b) While tip cells drive vessel elongation towards the source of VEGF and through EphB4/ephrinB2 signaling, tumor cells attract stromal cells. These stromal cells, comprising tumor-associated macrophages (TAM) and fibroblasts contribute to tumor angiogenesis through secretion of proangiogenic factors. During vessel elongation, new ECM is synthesized, and few pericytes will cover the neovessel. This—in conjunction with NO production and disruption of adherens junctions—results in a leaky vessel. (c) The resulting vasculature is tortuous with many dead ends and is prone to cell extravasation. Moreover, endothelial cells contribute to tumor growth by secreting supporting molecules in addition to carrying nutrients and oxygen.

*Vasculogenesis* and *angiogenesis* are two distinct processes, whereby the first represents vessel formation from differentiated precursors while the second originates from the preexisting vasculature. Capillaries formed during these processes are mainly constituted by endothelial cells which face the lumen of the vessel and sometimes, depending on their size, are surrounded by mural cells comprising pericytes and smooth muscle cells. Angiogenesis is essential during development while in adulthood the vasculature is usually quiescent, except during wound healing and the female reproductive cycle [1]. It is governed by several factors secreted by the targeted tissues and consists of four steps. The first step originates from the existing vessel from which the sprout arises. The combination of nitric oxide and Vascular Endothelial Growth Factor (VEGF) enhances vessel permeability by increasing capillary dilatation and leakiness, respectively. This allows extravasation of various plasma proteins which facilitate endothelial cell migration. Second, endothelial cells, to invade the hypoxic tissue, have to detach from the basement membrane they are laying on. This is mediated by the secretion of proteases such as proteins from the matrix metalloproteinase family (MMP-2, -3, -9) or through the inhibition of protease inhibitors such as TIMP (tissue inhibitor of MMP) proteins family. Third, after endothelial cells have detached, they proliferate and migrate to invade the hypoxic area, the source of proangiogenic factors, until they find contact to another capillary. During this process, as during axonal guidance, some cells lead the elongation of the sprouting vessel towards the angiogenic chemoattractant source. The endothelial cells forming the sprouting blood vessels exhibit distinct phenotypes. We can distinguish the tip cells at the leading edge of the sprout, stalk cells which follow the tip and phalanx cells which are quiescent cells from the mature vessel. Fate determination of the tip cells is dynamically regulated by VEGF-A/VEGFR1 and 2 and Notch/Dll-4 signaling pathways. Briefly, endothelial cells having the greater VEGFR2 to 1 ratio and higher Dll4 expression are more likely to adopt and keep the tip cell phenotype. VEGF-A-stimulated candidate cells will, therefore, signal through VEGFR2 which has a lower affinity for this ligand but a better protein kinase activity. VEGFR2 signaling leads to Dll-4 expression, activating Notch on the neighbour cell. Notch signaling in these endothelial cells leads to VEGFR1 upregulation while VEGFR2 is downregulated reducing the chance to become a tip cell. This system results in fate determination of the tip cells while keeping their neighbouring cells under a stalk cell phenotype; however, these phenotypes are not fixed over time [2]. First, the neighbourhood of tip cells is constantly changing due to endothelial cell migration, and this impacts on cells' VEGFR expression. Second, VEGFR2 to 1 ratio determines the time length of tip cell turnover, and, altogether, this result in an oscillatory Dll4 expression. Third, the main source of VEGF-A may differs during vessel elongation. After formation of tip cells, the sprout elongates through proliferation of stalk cells and reaches its target under the drive of tip cells. During the last step, cells start to differentiate and form a tube which will be stabilized through the recruitment of mural cells and secretion of extracellular matrix (ECM) [3]. An intact, functional vasculature requires a right balance between pro- and antiangiogenic factors; therefore, physiological angiogenesis is the result of a tightly controlled excess of proangiogenic factors. By contrast, tumor angiogenesis (Figure 1) originates from a disturbed balance between pro- and anti-angiogenic factors rendering endothelial cells unable to become quiescent and consequently support a constant growth of new blood tumor vessels [4]. In this work, we will focus on the following factors involved in tumor angiogenesis: VEGF/VEGFR, angiopoietins, and their receptors: Tie-1 and -2, Eph receptors, and their ephrins ligands, HIF1*α* (hypoxia-inducible factor 1 alpha), MMPs, and delineate their relationship with the important tumor-suppressor PTEN (phosphatase and tensin homologue).

## 2. Key Players in Angiogenesis

*VEGF* is the most important regulator of endothelial cell fate and acts as ligand of the VEGF receptor which belongs to the receptor tyrosine kinase (RTK) superfamily. VEGF is phylogenetically ancient: in *Drosophila* it is thought to be involved in blood cell positioning and comprises only one member; however, in mammals, 5 members of the VEGF family: VEGF-A, -B, -C, -D, and placenta growth factor: PLGF have been described. VEGF-A plays a prominent role in angiogenesis as shown by genetic studies of VEGF-A knock-out mice which die embryonically [5]. VEGF-A interacts preferentially with two of the three human members of VEGF receptors: VEGFR1 (Flt-1) and VEGFR2 (Flk1), while VEGF-B and PLGF bind to VEGFR1. Here we will not discuss in details VEGF-C and -D, which bind to VEGFR3 (Flt-4) and are involved in lymphangiogenesis. VEGF signaling through VEGFR1 and -2 results in an upregulation of proteases expression, recruitment of mural cells due to PDGF*β* secretion, increased vessel permeability, endothelial cell proliferation, migration, survival, and specialization [6]. 

*Angiopoietin* (Ang) ligands bind to *Tie* receptors, which are single transmembrane RTK. These proteins possess three immunoglobulin-like (Ig-like) domains, three epithelial growth factor domains (EGF) and three fibronectin type III domains giving the name Tie: Tyr-kinase with Ig and EGF homology domains. The two isoforms of Tie: Tie-1 and -2 are involved in vascular development as demonstrated by loss-of-function studies [7, 8]; however, Tie-2 is much more studied than its isoform. Tie-2 is required early in embryonic development for plexus remodeling and maturation. Tie-2 is predominantly expressed on endothelial cells and principally binds to Ang-1 and -2 which are agonist and antagonist ligands, respectively. Ang-1 stimulation of Tie-2 leads to endothelial cell survival and migration, limits vessel permeability, and stimulates smooth muscle cells recruitment. Tie-2 signaling triggers a negative feedback loop involving the forkhead transcription factor FOXO1 and Ang-2 expression [9]. 

Another system of receptor/ligand involved in angiogenesis regulation is the Eph/ephrin couple. *Eph receptors* (first cloned from an erythropoietin producing hepatocellular carcinoma cell line) belong to the RTK superfamily and are involved in embryogenesis, axonal guidance, and angiogenesis [10–12]. Eph receptors are divided into two subfamilies: A and B, which generally bind to ephrin A and B ligands, respectively. In contrast to the other RTK whose ligands are secreted molecules, ephrin ligands are membrane bound. This renders this couple quite unique as (a) it requires two neighbouring cells to signal, and (b) numerous studies have demonstrated that this signal is transmitted by both receptor and ligand. This bidirectional signaling is involved in the regulation of the cytoskeleton leading to modulation of cell adhesion, repulsion, and motility and contributing to cell positioning [13]. Wang et al. first showed that EphB4 and ephrinB2 are key players in the development of the vascular system by the use of transgenic mice. They demonstrated that EphB4 is restricted to veins while ephrinB2 is expressed only by arteries, and a lack of one of these actors is lethal due to perturbed arteriovenous differentiation [14]. Indeed ephrinB2 reverse signaling was demonstrated to participate to tip cell guidance through control of VEGFR2 signaling guarantying a successful vasculature formation [15]. Moreover, ephrinB2 was shown to contribute to vessel maturation as it is expressed on mesenchymal cells such as smooth muscle cells, and its binding to EphB leads to mural cells recruitment [16].

As angiogenesis responds to an increased need in oxygen, hypoxia is a crucial part of the process. The main effector of the adaptive response to hypoxia is the transcription factor HIF1. HIF1 is a member of the basic helix-loop-helix/PAS protein transcription factor family and comprises HIF1*α* and HIF2*α*, which to be active require heterodimerization with HIF1*β* [17]. The *α* subunit acts as an oxygen sensor as it is degraded under normoxia while the *β* subunit is constitutively expressed. In normoxic condition, the prolyl hydroxylase enzymes (PHD1-3) are in an active state and hydroxylate HIF1*α* on two proline residues (402 and 564) within its oxygen-dependent degradation domain (ODD) [18]. Hydoxy-HIF1*α* is recognized by the complex formed by the ubiquitin ligase VHL (von Hippel-Lindau) and the elongin proteins, and this binding triggers the rapid HIF1*α* degradation through the proteasomal pathway. In contrast, under hypoxic conditions, HIF1*α* forms a heterodimer with HIF1*β*, and their translocation to the nucleus acts on gene expression through their binding to the hypoxia response element (HRE). Numerous genes are regulated by HIF1, some related to red blood cell production (erythropoietin), some to the vascular architecture and tone (VEGF, VEGFR1, or Nitric Oxide Synthase 2), some to the metabolism (Glut-1 and Glut-3), and some to cellular proliferation and differentiation (TGF*β*, Cyclin G2, or p21) [19]. In 2005 our group demonstrated an upregulation of EphB4, ephrinB2, EphA2, ephrinA1 mRNA and protein expression in hypoxic tissues [20]. 

Finally, one of the major sources of angiogenic factors is the ECM, first because it can store growth factors such as VEGF, second because degradation of some of its component leads to the formation of pro- or anti-angiogenic factors. Among the proteases involved in the release of such factors, MMP proteins which are members of a zinc-dependent family of endopeptidases and more precisely MMP2 and 9 are key players. MMP9 was shown to be activated in numerous cancers and to contribute to tumor angiogenesis by increasing the bioavailability of VEGF, basic fibroblast growth factor (bFGF) by degrading collagen types IV, XVIII and perlecan [21, 22]. Moreover, MMPs are involved in cell migration by interfering with the cell-ECM and cell-cell interactions.

## 3. Tumor Angiogenesis

All the factors mentioned above are not only involved in physiological but also in tumor angiogenesis. Tumor development is first limited to 1-2 mm^3^ due to the lack of oxygen, nutrients, and growth factors. This restriction may be the cause of tumor latency until the so-called *angiogenic switch* occurs. Angiogenic switch is defined as the acquisition by tumor cells of properties required for their unopposed growth, where the balance is skewed towards a proangiogenic phenotype [28]. As tumor cells are in a hypoxic state prior to the angiogenic switch, the HIF1 pathway is constitutively active and leads to the expression of angiogenesis-related genes (VEGF/VEGFR, MMPs, Eph/ephrins). Noteworthy, even after the tumor vasculature is fully developed and functional, most of the tumor cells still maintain an activated HIF1 pathway [29]. As mentioned earlier, HIF1 target genes will lead to endothelial cell recruitment to the tumor site mainly through VEGF/VEGFR activity. This effect is amplified by tumor cells, which also have the capability to attract stromal cells as cancer-associated fibroblasts (CAF) [30] and tumor-associated macrophages (TAM) [31]. TAMs seem to play a pivotal role as they secrete VEGF, MMP9, and even immunosuppressive molecules. The resulting vessels are often dilated due to VEGF and leaky because of nitric oxide overproduction and deficient mural cell recruitment [32]. Tumor vessels are characterized by their irregular and tortuous shape consecutive to aberrant endothelial cell proliferation and often possess dead ends leading to a higher risk of hemorrhage. Therefore, a high tumor-associated microvascular density does not reflect high oxygen level in the tumor as blood flows only irregularly through tumor vessels, leading to hypoxic areas within the tumor. Beside the angiogenic factors directly depending on HIF1 pathway, Ang-2/Tie and Eph/ephrins receptor/ligand pairs have also been shown to play a role during the angiogenic switch. Cancer-dependent Ang-2 upregulation occurs principally in tumor-associated endothelial cells and can be used as a biomarker of tumor progression [33]. High levels of Ang-2 trigger endothelial cells apoptosis mediating vessel pruning. This leads to the formation of hypoxic area which in turn upregulates VEGF expression and results in higher vascular density [34]. The roles of EphB4 and ephrinB2 expressed on blood vessels are more controversial. Although several reports showed that EphB4 stimulation impairs tumor growth due to a defect in tumor angiogenesis [35, 36], Kumar et al. demonstrated the opposite [37–39]. Concerning ephrin reverse signaling, Martiny-Baron et al. demonstrated that blocking ephrinB2 stimulation by the use of monomeric soluble EphB4 impaired tumor growth in nude mice and correlates with a decreased microvessel density [40].

## 4. PTEN and Tumor Angiogenesis

**Figure 2:**
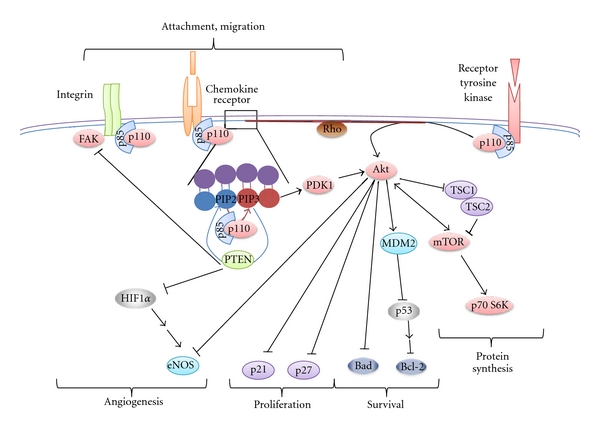
The PI3 kinase pathway and PTEN.

A remarkable property of tumor angiogenesis is that all involved factors described earlier signal through the PI3 Kinase (PI3K) pathway [41–44]. The PI3K pathway is a signaling route involved in many cellular processes such as cell survival, proliferation, or migration. The PI3K protein class IA is activated in response to RTK stimulation, while the class IB is activated by G-protein-coupled receptors [45]. PI3K activation leads to the transformation of phosphatidylinositol-4,5-bisphosphate (PIP2) into phosphatidylinositol-3,4,5-trisphosphate (PIP3). This in turn will activate Akt, a serine-threonine protein kinase which has numerous targets comprising the mammalian target of rapamycin (mTOR), a protein complex stimulated in hypoxic and nutrient-poor environment. The major regulator of this signaling pathway is PTEN, also known as MMAC1 (Mutated in Multiple Advanced Cancer1) or TEP1 (TGF*β*-regulated and epithelial cell-enriched phosphatase). PTEN gene in human locates on chromosome 10q23.3 and encodes protein of 40–50 kilodaltons as in many organisms, with the exception of PTEN from *Caenorhabditis elegans*. This protein comprise 4 domains: at the N terminal a PIP2 binding site, then a phosphatase domain, a C2 domain containing phosphorylation sites, followed by a PDZ binding motif at the C-terminal end [46]. PTEN acts as a phosphatase on both lipids and proteins; it antagonizes PI3K pathway by transforming PIP3 into PIP2 (Figure 2) [47] and dephosphorylates proteins such as SHC or FAK [48, 49]. Several studies also report a role for PTEN in cell migration, independent of its phosphatase activity as the expression of a truncated form of PTEN possessing only the PTEN C2 domain inhibits cell migration [50, 51]. PTEN is regulated posttranscriptionally by miRNA, such as miR-21 [52] and posttranslationally through phosphorylation, acetylation, ubiquitylation, or by regulation of its localization [53]. As the PI3K pathway is activated during angiogenesis, PTEN can be considered as a major intracellular regulator of this process.

Before discussing the role of PTEN in tumor endothelial cells, we will first summarize its functions in cancer cells. PTEN is frequently found deleted, mutated, or downregulated in human malignancies. PTEN mutation primarily affects the PTEN phosphatase domain and leads to several diseases including Cowden's and the Bannayan-Riley-Ruvalcaba syndrome, the Lhermitte-Duclos disease, as well as an increased risk of breast, thyroid, and endometrial cancers (Table 1). 

Regarding HIF1, PTEN was found to inhibit its stabilization and its transcription factor activity in glioblastoma cell lines [54]. Moreover, several studies report a concomitant loss of VHL and PTEN, two important regulators of the HIF1 pathway, in clear cell renal cell carcinoma [55, 56]. Since HIF1 signals through PI3K pathway, leading to an increased VEGF expression, PTEN loss in cancer cells leads to an increased VEGF expression due to upregulation of HIF1. This was shown by several investigators using different models: Fang with PC-3 cells [57], Tian with HepG2 cells [58]. Takei et al. [59] demonstrated that PTEN reintroduction led to a decreased HIF1-*α*, VEGF, and PCNA expression in ovarian cancer cells. As to MMPs, a study using a multiple myeloma cell line and cells originating from patients showed that PTEN transfection results in a decreased mRNA and protein expression of MMP2, MMP9 and FAK (Focal Adhesion Kinase), leading to decreased cell migration [60]. Furthermore, microarray analysis of gastric carcinomas highlighted a negative correlation between PTEN expression and VEGF, MMP2, and MMP9 expression, and the authors concluded that PTEN has an inhibitory effect on microvascular density [61]. PTEN loss at post-transcriptional level is also involved in tumor angiogenesis. Giovannetti et al. [62] reported that in pancreatic ductal adenocarcinoma, miR-21 is responsible for elevated expression of MMP2, MMP9, and VEGF and this could be abrogated by treatment of the cells with a PI3K inhibitor. As miR-21 is overexpressed in many tumors, this strategy may represent a valuable tool to control tumor angiogenesis [63]. Concerning the Ang/Tie system, it was shown that Ang-1 stimulation of Tie-2 triggers PI3K pathway activation [64]. Moreover, Findley et al. described a Tie2 receptor shedding which is able to bind Ang-1 and -2 and consequently inhibits Tie2 phosphorylation and activation [65]. This process is regulated by VEGF-dependent activation of PI3K pathway, and PTEN overexpression increases Tie-2 shedding. 

PTEN localization within the cell is also part of PTEN regulation, since defective PTEN translocation to the cell membrane impairs its control of PI3K signaling [87]. This was shown by Molina et al., who found that the adaptor protein NHERF1 is involved in PTEN translocation to the cell membrane [88]. They proved that impaired PTEN positioning leads to sustained Akt activation. This can be of primarily importance as NHERF1 has been shown to interact with several RTK such as PDGFR*β* and EGFR [89, 90], which all play a role in tumor angiogenesis. Moreover, PTEN translocation to the cell membrane is not solely involved in regulation of RTK's downstream signaling, but is also emerging as a modulator of their expression [91]. Recent work from our lab has shown that PTEN also interacts with EphB1 signaling (manuscript submitted). As mentioned above, Eph receptors and ephrins are frequently overexpressed by cancer and tumor endothelial cells, and this may lead to an enhanced tumor angiogenesis. Among the various pathways activated by Eph receptors, PI3K has been reported in a number of studies [13, 92]. Moreover, Brisbin showed a direct linkage between VAB-1 and DAF-18, the *C. elegans* form of Eph and PTEN, respectively [93], an interesting observation in line with our findings. As during tumor angiogenesis both cancer and endothelial cells are involved, PTEN status in endothelial cells is also crucial. Many studies have shown that PTEN expression can be disturbed in both stromal cells and endothelial cells [94, 95]. This can be primordial as reduced PTEN signaling in endothelial cells as well as PI3K activation leads to an enhanced cell proliferation, survival, and migration—all important features for angiogenesis [96–98]. Moreover, tumor vessels are characterized by their inability to become quiescent, and it was recently shown that PTEN is involved in endothelial cell aging [99]. 

Interestingly, PTEN is susceptible to oxidation. As highly proliferative cells overproduce reactive oxygen species (ROS), this mechanism may account for the reduction of PTEN levels in cancer and cancer-related cells [100]. A vicious circle, therefore, begins as cancer cells produce more ROS, affecting PTEN level and enhancing VEGF secretion [101, 102]. VEGF in turn acts on endothelial cells and stimulates ROS production mainly by NOX1 (NADPH Oxydase 1) [103], potentially affecting PTEN level as well as cell proliferation. VEGF will also amplify endothelial cell migration and contribute to the angiogenic switch enhancing tumor growth [104]. Another derivative of oxygen is NO, which also plays a role in angiogenesis as it contributes to vessel dilatation. Church et al. demonstrated that PTEN is involved in endothelial Nitric Oxide Synthase regulation in both cancer and endothelial cells [105] and should, therefore, affect tumor angiogenesis. 

Cancer cells have a supportive effect on endothelial cells; however, the reverse is also true, with the formation of the so-called vascular niche. Koistinen et al. demonstrated that VEGF-stimulated endothelial NO production maintains Acute Myeloid Leukemia (AML) cell growth, and this involved the PI3K pathway [106]. Several examples of endothelial cells stimulating solid tumor growth have also been described. VEGF promotes endothelial cell survival through PI3K activation and increased expression of the anti-apoptotic protein Bcl-2, and this can be antagonized by an increased PTEN expression [107]. Recently, it was shown that Bcl-2-expressing endothelial cells also produce IL-8, which acts on tumor cells and leads to an increased invasiveness and metastatic ability [108]. Finally Park et al. demonstrated that Nerve Growth factor (NGF) stimulates endothelial cells to produce MMP2, a process inhibited by PTEN transfection [109].

## 5. Anti-tumor Therapies and PTEN

It now clearly appears that PTEN is involved in all the differrent steps leading to tumor angiogenesis. As a consequence, although the PI3K pathway is considered as the major signaling node in physiological and tumor angiogenesis, PTEN can be seen as the main intracellular antagonist to this process. Many efforts have been made to control tumor angiogenesis and related tumor growth, but here again PTEN status appears to be critical [110, 111]. Initial strategies to restrict tumor-associated angiogenesis used anti-VEGF compounds such as monoclonal antibodies against VEGF-A (Bevacizumab) or VEGFR tyrosine kinase inhibitors (Sorafenib, Sunitinib). However, after promising results in clinical trials, tumor resistance to these treatments has emerged as a major problem. These resistances are often related to a switch from VEGF- to bFGF- or EGF-dependent angiogenesis and require the HIF-1*α* and PI3K pathways signaling [112–114]. Many studies have now confirmed that resistance to anti-EGFR therapies is often linked to PTEN status in cancer cells, and PTEN loss negatively correlates with clinical response to these therapies [115, 116] or others (Table 2). This problem can be overcome by the use of combined therapies which show synergistic effects and are now tested in clinical trials [117, 118]. MMPs overexpression is another resistance mechanism. We previously discussed the negative correlation between PTEN and MMPs expression; therefore, cancer cells with reduced or lost PTEN activity are more prone to develop this kind of resistance. To avoid the emergence of such resistance, different strategies aiming at antagonizing the PI3K pathway or reintroducing the expression and/or activity of PTEN have been tried. Many PI3K inhibitors, as well as Akt and mTOR inhibitors, are now being tested in clinical trials [119, 120]. Although there are numerous inhibitors of the PI3K pathway, to date no PTEN inducer has been found, explaining why only PTEN transfection or gene therapy has been tried. Transfection of wild-type PTEN into human prostate cancer cells sensitizes cells to radiation and leads to a decreased tumor-induced angiogenesis [121]. In this work, both cancer- and tumor-associated endothelial cells were affected by this treatment. As previously mentioned, PTEN reintroduction decreases HIF-1*α* and VEGF levels [57–59], and Lee et al. showed promising results using this strategy in mice [122]. As PI3K inhibitors monotherapy has often shown mitigated results and gene therapy experiments are just emerging, PTEN-stimulating molecules are urgently needed. The greatest challenge, however, remains the difficulty to set up a robust PTEN activation model, where molecular stimulators of PTEN could be tested.

## 6. Concluding Remarks

A myriad of studies have now demonstrated the central role of the PI3K pathway in tumorigenesis and angiogenesis. Overactivation of this pathway, a common event in cancer cells, leads to enhanced and uncontrolled tumor angiogenesis and can be antagonized by the phosphatase and tumor-suppressor PTEN. Moreover, PTEN is involved in the control of cell proliferation, migration, and survival of both cancer and tumor-associated endothelial cells. Ideally, combining PTEN-targeted therapy to classical chemotherapy should be a promising strategy, leading to (a) normalization of the tumor vasculature, (b) sensitization of cancer cells to further chemo- and radiotherapy, and (c) avoidance of resistance to such treatments. Further progress and development of PTEN-targeted therapy are, therefore, required and may lead to improvement of the current anticancerogenic strategies.

## Figures and Tables

**Table 1:** PTEN-related diseases and associated cancer susceptibility. Percentages are indicated when available.

Disease	PTEN defect	Clinical symptoms	Cancer susceptibility
Cowden's syndrome [23, 24]	Splice variants	Trichilemmonas, macrocephaly, papillomatous papules	Breast (65%), thyroid (75%), endometrium (5–10%)
	Deletion: coding sequence		
	promoter (10%)		
	Nonsense mutation		
	Missense mutation (85%):		
	C124: no phosphatase activities, G129: no lipid phosphatase activity, K289: no nuclear translocation		

The Bannayan-Riley-Ruvalcaba syndrome [23, 24]	Deletion (11%)	Macrocephaly, intestinal polyposis, developmental delay, lipomas, speckled penis in male	Breast, thyroid, endometrium, rare colorectal carcinoma
	Nonsense mutation		
	Missense mutation (60%)		


The Lhermitte-Duclos disease [25]	Splice variants	Ataxia, increased intracranial pressure, seizures	Not demonstrated
	Deletion		
	Nonsense mutation		
	Missense mutation (80%)		

Proteus/Proteus-like syndrome [26]	Missense mutation (20 and 50%, resp.)	Epidermal nevus, disproportionate overgrowth of the skull, limbs, vertebrate, Lipomas, vascular malformation	rare events: cystadenoma of the ovary, testicular tumors, central nervous system tumors, parotid monomorphic adenomas

Autism [27]	Missense mutation (around 10%)	Sometimes associated with macrocephaly	Not demonstrated

**Table 2:** Clinical trials having shown an impact of the PTEN status on the response to cancer treatment.

Type of cancer	Metastatic form	Treatments	References
Colorectal		Cetuximab, panitumab	[66–69]
	×	Cetuximab (+irinotecan)	[70–72]

Breast		Trastuzumab, lapatinib	[73–76]
	×	Trastuzumab	[76–78]
		Endocrine therapy	

Glioblastoma		Gefitinib, erlotinib	[79–81]
		Erlotinib + temozolomid	

Gastric		Streptozotocin, doxorubicin, 5-fluorouracil, etoposide/cisplatinum	[82, 83]
	×	Streptozotocin, doxorubicin	[82]

Lung		Gefitinib, erlotinib	[84, 85]
	×	Gefitinib, erlotinib	[85]

Pancreas		Gemcitabine	[62]

Esophageal		5-fluoropyrimidine, taxane, platinum, PI3K pathway inhibitor	[86]
